# Mitochondrial Genome-Knockout Cells Demonstrate a Dual Mechanism of Action for the Electron Transport Complex I Inhibitor Mycothiazole

**DOI:** 10.3390/md10040900

**Published:** 2012-04-16

**Authors:** Kirsten J. Meyer, A. Jonathan Singh, Alanna Cameron, An S. Tan, Dora C. Leahy, David O’Sullivan, Praneta Joshi, Anne C. La Flamme, Peter T. Northcote, Michael V. Berridge, John H. Miller

**Affiliations:** 1 Centre for Biodiscovery and Schools of Biological Sciences, Victoria University of Wellington, PO Box 600, Wellington, New Zealand; Email: kirstenjoymeyer@hotmail.com (K.J.M.); dora.leahy@gmail.com (D.C.L.); david.osullivan@vuw.ac.nz (D.O.); praneta.joshi@spcorp.com (P.J.); anne.laflamme@vuw.ac.nz (A.C.L.F.); 2 Centre for Biodiscovery and School of Chemical and Physical Sciences, Victoria University of Wellington, PO Box 600, Wellington, New Zealand; Email: jonathan.singh@vuw.ac.nz (A.J.S.); peter.northcote@vuw.ac.nz (P.T.N.); 3 Malaghan Institute of Medical Research, Wellington, New Zealand; Email: acameron@malaghan.org.nz (A.C.); antan@malaghan.org.nz (A.S.T.); mberridge@malaghan.org.nz (M.V.B.)

**Keywords:** metabolic inhibitor, mitochondrial electron transport complex I, mycothiazole, natural product, reactive oxygen species

## Abstract

Mycothiazole, a polyketide metabolite isolated from the marine sponge *Cacospongia mycofijiensis*, is a potent inhibitor of metabolic activity and mitochondrial electron transport chain complex I in sensitive cells, but other cells are relatively insensitive to the drug. Sensitive cell lines (IC_50_ 0.36–13.8 nM) include HeLa, P815, RAW 264.7, MDCK, HeLa S3, 143B, 4T1, B16, and CD4/CD8 T cells. Insensitive cell lines (IC_50_ 12.2–26.5 μM) include HL-60, LN18, and Jurkat. Thus, there is a 34,000-fold difference in sensitivity between HeLa and HL-60 cells. Some sensitive cell lines show a biphasic response, suggesting more than one mechanism of action. Mitochondrial genome-knockout ρ^0^ cell lines are insensitive to mycothiazole, supporting a conditional mitochondrial site of action. Mycothiazole is cytostatic rather than cytotoxic in sensitive cells, has a long lag period of about 12 h, and unlike the complex I inhibitor, rotenone, does not cause G_2_/M cell cycle arrest. Mycothiazole decreases, rather than increases the levels of reactive oxygen species after 24 h. It is concluded that the cytostatic inhibitory effects of mycothiazole on mitochondrial electron transport function in sensitive cell lines may depend on a pre-activation step that is absent in insensitive cell lines with intact mitochondria, and that a second lower-affinity cytotoxic target may also be involved in the metabolic and growth inhibition of cells.

## 1. Introduction

**Figure 1:**
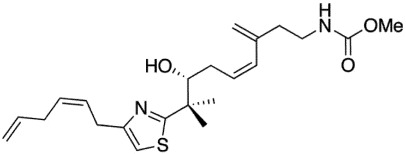
Structure of mycothiazole.

Mycothiazole (MYZ, Figure 1) is a polyketide heterocycle first isolated from the marine sponge *Spongia mycofijiensis* in 1988 [1] and shown to be anti-helminthic and toxic to mice. Structural analog studies revealed that the thiazole component of MYZ was important for its bioactivity [2]. The original chemical structure of MYZ as proposed by Crews *et al.* [1] was later found to be incorrect, and a corrected structure was subsequently published in 2006 [3]. Screened against the NCI panel of 60 cancer cell lines, MYZ displayed a broad range of activities in different cell lines with potent nanomolar activity against small (SCLC) and non-small (NSCLC) cell lung cancer cell lines [4].

MYZ was shown to inhibit hypoxia-inducible factor-1 (HIF-1) by Nagle and colleagues [5]. Later work by the same group showed MYZ to be an inhibitor of complex I (NADH-ubiquinone oxidoreductase) of the mitochondrial electron transport (MET) chain [6]. Using a digitonin-permeabilized human breast tumor T47D cell line, MYZ was shown to act like rotenone in inhibiting oxygen consumption by the cells following addition of malate/pyruvate. The block by MYZ was completely reversed by addition of succinate, which bypasses complex I and passes electrons directly to complex II. MET chain inhibitors such as rotenone (CI), malonate (CII), antimycin A (CIII), and cyanide (CIV) act by direct inhibition of the protein complexes, or by acting as electron acceptors and disrupting the chain [7]. Reactive oxygen species (ROS) are largely produced at complexes I and III [8], but ROS can also be produced from complex II [9]. Complex I inhibitors, by altering mitochondrial function, are potential cancer chemotherapeutic agents because of the links of mitochondria to apoptosis and their effects on bioenergetics and ROS production [10,11]. Thus, the National Cancer Institute (NCI) carried out a data mining screen for potential novel complex I inhibitors, and of 10 compounds selected for possible activity, 5 showed good inhibition of complex I [10]. Complex I inhibitors, like rotenone and the annonaceous acetogenin bullatacin, for example, bind to a 30 kDa protein that is part of the DN1 subunit of complex I [11]. Bullatacin also inhibits the NADH oxidase found in the plasma membrane of tumor cells that allows the cells to produce ATP in the hypoxic environment of the tumor; thus, this complex I inhibitor blocks both oxidative and anaerobic ATP and nucleotide production.

In addition to MET, a trans-plasma membrane electron transport (tPMET) system exists consisting of NADH oxidases and ubiquinone that makes use of extracellular, rather than intracellular, oxygen [12,13]. This system has been most clearly demonstrated in cells that lack mitochondrial DNA (ρ^0^ cells) [14]. These ρ^0^ cells have no functional MET since 13 subunits of the four MET complexes are encoded in the mitochondrial genome [15]. 

The purpose of the present study was to investigate the action of MYZ in different cancer cell lines and to determine the effect of the drug on cellular bioenergetics using ρ^0^ cells that lack mitochondrial DNA and therefore have no functional complex I. This approach has the potential to unmask a secondary mode of action of MYZ that could explain a biphasic activity seen in some cells and may provide information on its nanomolar mechanism of action in sensitive cells and its micromolar activity in resistant cells.

## 2. Results and Discussion

### 2.1. Mycothiazole Isolation and Purification

Mycothiazole (MYZ) (Figure 1) was isolated and purified from the Tongan marine sponge *Cacospongia mycofijiensis* as described in the experimental section. Four other compounds—latrunculin A, dendrolasin, laulimalide, and zampanolide—were also isolated from the same sponge species [16].

### 2.2. Effects of MYZ on Cell Metabolism and the Proliferative Response

**Figure 2:**
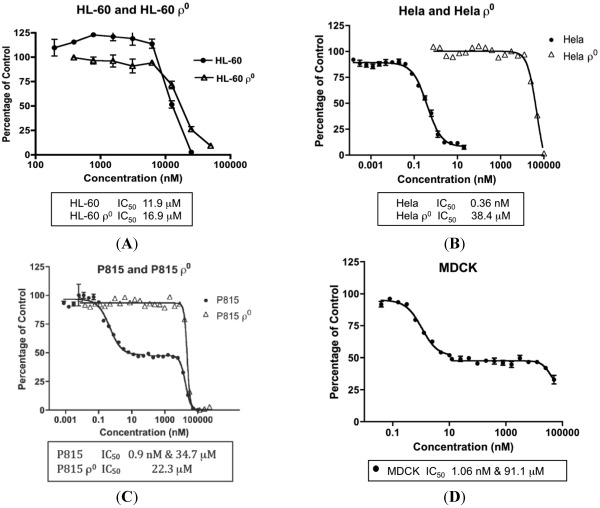
Concentration-dependent effects of Mycothiazole (MYZ) on selected cell lines. Representative graphs of 48 h 3-(4,5-dimethylthiazol-2-yl)-2,5-diphenoyltetrazolium bromide (MTT) assays with MYZ in HL-60/HL60ρ^0^ cells (**A**), HeLa/HeLaρ^0^ cells (**B**), P815/P815ρ^0^ cells (**C**), and MDCK cells (**D**). The IC_50_ values are given at the bottom of each graph (*n* = 3 or more independent experiments).

**Figure 3:**
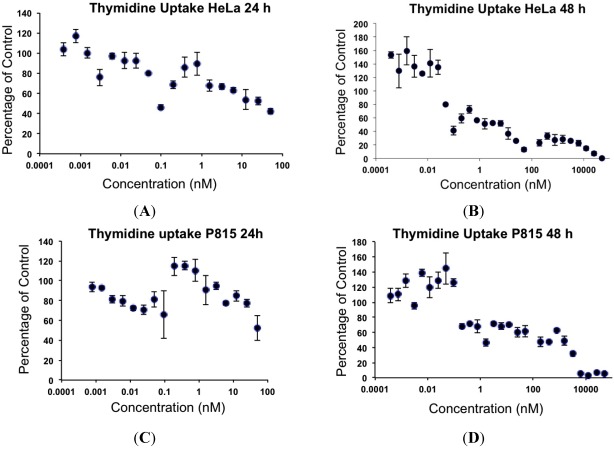
Effects of MYZ on ^3^H-thymidine uptake by HeLa and P815 cells after 24 h and 48 h exposure. Representative graphs are shown from two independent experiments. HeLa (**A**, **B**) and P815 (**C**, **D**) cells were exposed to 50 nM MYZ for 24 h (A, C) and 48 h (B, D), and ^3^H-thymidine was added during the last 8 h of culture.

The concentrations of MYZ that produced 50% inhibition (IC_50_) of MTT reduction for cell lines or CSFE proliferation for T cells were determined using eleven established cell lines and primary murine splenic T-cells (Table 1). These cells showed diverse responses to MYZ, with most cell lines (HeLa, P815, HeLaS3, 143B, B16, 4T1, MDCK, and RAW264.7) and the murine T-cells being highly sensitive, and three cell lines (HL-60, LN18 and Jurkat) being relatively resistant to the drug. For example, HL-60 cells had a mean IC_50_ value of 12 μM; whereas, HeLa were highly sensitive with a mean IC_50_ of 0.36 nM (Table 1, Figure 2A,B). In contrast, P815 and MDCK cells exhibited a biphasic response (Table 1, Figure 2C,D); thus, low nanomolar concentrations of MYZ inhibited MTT responses of these cells by about 50%; whereas, more extensive inhibition was observed at micromolar concentrations, suggesting both high and low-sensitivity mechanisms of action. Preliminary attempts to investigate cell recovery by washing out MYZ for 24 h after a 24 h exposure showed that some cell lines (e.g., 143B) remained sensitive after washout, others (P815) were partially reversed in their ability to proliferate, and some, like HeLa, completely recovered from the effects of the drug (data not shown). ^3^H-thymidine uptake was used to confirm that the MTT assay was measuring a proliferative response and not just metabolic depression of non-dividing cells (Figure 3). 

**Table 1:** Summary of MYZ effects on cellular MTT and carboxyfluorescein succinimidyl ester (CFSE) responses. IC_50_ values were determined by MTT reduction at 48 h in different cell lines and by the CFSE proliferation assay for the T cells. Note the biphasic response of P815 and MDCK cells.

Parental Cell Lines	IC_50_ (Mean ± SEM)	*n*	ρ^0^ Cell Lines	IC_50_ (Mean ± SEM)	*n*
***Sensitive***					
HeLa	0.36 ± 0.09 nM	4	HeLaρ^0^	78.4 ± 20.2 μM	3
HeLa S3	1.84 nM	2			
143B	3.24 ± 1.6 nM	4	143Bρ^0^	50.7 μM	1
B16	13.8 ± 3.6 nM	3	B16ρ^0^	34.7 μM	1
4T1	12.1 ± 2.5 nM	3			
RAW 264/7	1.54 nM	2			
PrimaryT Cells	CD4 2.04 ± 0.37 nMCD8 7.20 ± 0.52 nM	55			
***Biphasic***					
P815	(a) 1.02 ± 0.22 nM (b) 30.3 ± 5.1 μM	45	P815ρ^0^	36.4 ± 9.2 μM	3
MDCK	(a) 1.47 ± 0.79 nM (b) 91.1 μM	31			
***Insensitive***					
HL-60	12.2 ± 2.2 μM	5	HL-60ρ^0^	26.1 ± 9.2 μM	2
LN18	26.5 ± 3.1 μM	3			
Jurkat	26.5 μM				

MYZ has been shown to inhibit complex I of MET, and therefore cell lines lacking MET were used to investigate how this would effect the MYZ potency. Ablation of MET function in HL-60 cells by mitochondrial genome deletion (HL-60ρ^0^ cells) had little effect on the IC_50_ value for MYZ (26 μM compared to 12 μM in the parental cell line) (Table 1, Figure 2A). On the other hand, the IC_50_ for MYZ with HeLaρ^0^ cells (Table 1, Figure 2B) was 78 μM compared to 0.36 nM in the parental cell line—a 217,000-fold decrease in sensitivity in the absence of MET function. In all cases, ρ^0^ cells were highly insensitive to the action of MYZ with IC_50_ values in the 26–78 μM range. In P815ρ^0^ cells the initial 50% reduction in MTT response to nanomolar concentrations of MYZ was lost. An IC_50_ value of 22 μM was observed, the MTT reduction occurring in the same concentration range as the micromolar part of the biphasic response in P815 cells (17 μM).

### 2.3. Effects of MYZ on Cell Viability

**Figure 4:**
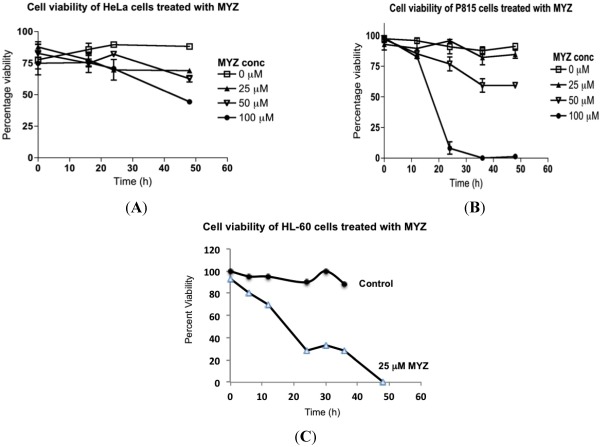
Effects of MYZ on cell viability of HeLa, P815, and HL-60 cells. Cells were exposed to MYZ for various times, and the percent viability was determined by trypan blue dye exclusion. HeLa cells (**A**) and P815 cells (**B**) were exposed to MYZ at concentrations ranging from 0–100 μM. HL-60 cells were treated with vehicle or 25 μM MYZ (**C**). Results are representative of 3 or more independent experiments.

To distinguish cytotoxic from cytostatic effects, trypan blue dye exclusion was used with HeLa, P815, and HL-60 cells (Figure 4). MYZ treatment at concentrations as high as 25 μM had little effect on the viability of HeLa and P815 cells, even after 48 h incubation with the drug. With HeLa cells, 50% cytotoxicity was seen after 48 h exposure to 100 μM MYZ (Figure 4A), and with P815 cells, 25–30 h exposure to 50 μM MYZ resulted in 40% loss of viable cells (Figure 4B). Thus, the cytotoxic effects of MYZ were only seen at concentrations four or more orders of magnitude above the IC_50_ concentration determined in the MTT assay. In contrast, treatment of HL-60 cells with MYZ at a concentration comparable to its IC_50_ in the MTT assay (25 μM) resulted in complete loss of viability (Figure 4C). Cytotoxic effects of MYZ with HL-60 cells were evident as early as 5 h following exposure.

### 2.4. Lag before MYZ Exerts Its Effects on Cell Metabolism

**Figure 5:**
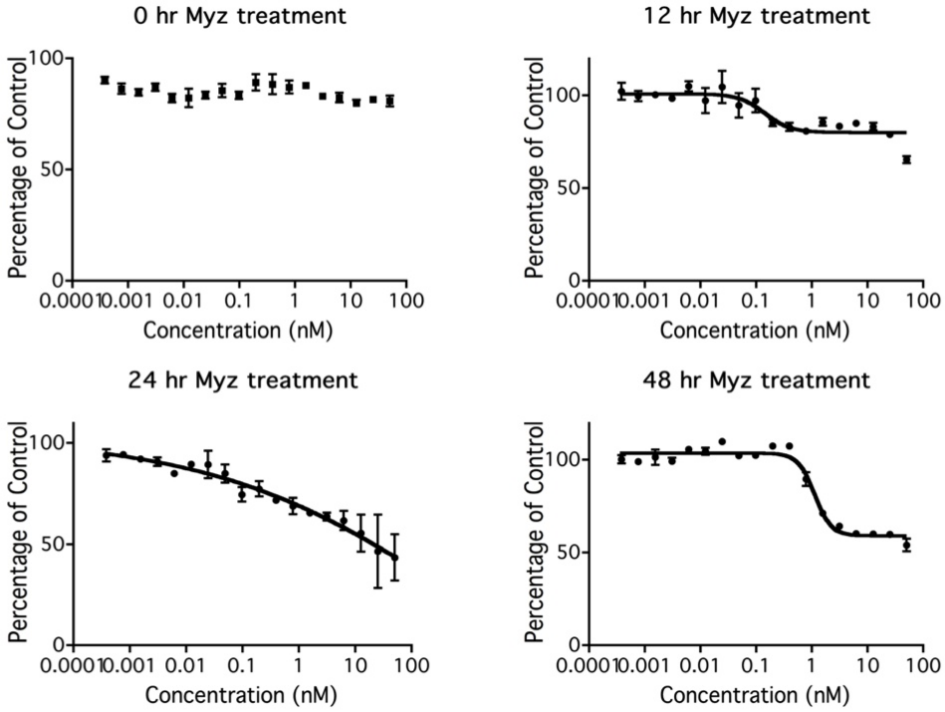
Effect of time of exposure to MYZ on MTT reduction by P815 cells. Representative graphs are presented of MTT assays after different MYZ exposure times of 0 h, 12 h, 24 h, and 48 h. For the 0 h treatment, MTT reagents were added immediately after the MYZ was added to the cells. Results are representative of two independent experiments.

To investigate the kinetics of metabolic inhibition by MYZ, MTT assays were performed on P815 cells treated with MYZ for 0, 12, 24, and 48 h (Figure 5). There was an unexpected lag period of 12 h before the MTT response began to be affected by MYZ. The threshold concentration required to give an effect on metabolism was about 0.1 nM MYZ. As expected, inhibition at each concentration increased with longer exposure times. 

### 2.5. Effects of MYZ on the Cell Cycle

**Figure 6:**
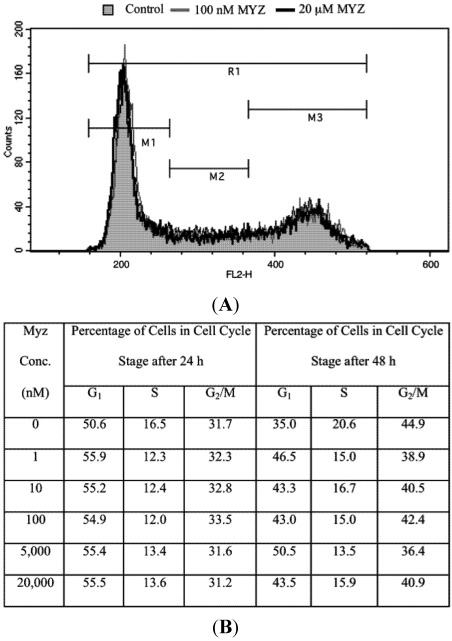
Lack of effect of MYZ on cell cycle of HeLa cells after 24 and 48 h exposure. (**A**) Representative cell cycle distribution after 24 h treatment with MYZ at concentrations of 0 nM (Control; gray shaded area), 100 nM (gray line), and 20 μM (black line); (**B**) Summary table of cell cycle analysis of HeLa cells treated with varying concentrations of MYZ at 24 h and 48 h. The average % for two independent experiments is presented for MYZ concentrations of 0–100 nM. The 5 and 20 μM concentrations are from a single determination.

Using flow cytometry of PI-stained cells, the effects of MYZ on progression of HeLa cells through the cell cycle were investigated. MYZ after 16 h exposure had no effect on cell cycle progression at concentrations up to 100 nM, three orders of magnitude greater than the MTT IC_50_, (Table 2). Synchrony of HeLa cells with 2 mM thymidine block, then release also showed no significant changes in cell cycle distribution (data not shown). A similar lack of a cell cycle effect was seen with HL-60 cells at concentrations from 0.1–50 μM (*n* = 3 preparations) (Figure S2 of Supplementary Data). To ensure a block was not missed in the HeLa cells, concentrations up to 20 μM and exposures up to 48 h were also tested, and again, no treatment resulted in cell cycle arrest (Figure 6).

**Table 2:** Lack of MYZ effect on the cell cycle of HeLa cells after 16 h exposure. HeLa cells were treated with 0, 0.1, 1, 10, and 100 nM MYZ for 16 h, and the % of cells in each phase of the cell cycle was determined (*n* = 4–7 independent experiments). None of the G_2_/M values were significantly different from the control G_2_/M value (One-way ANOVA, Dunnett’s multiple comparison test).

Concentration MYZ (nM)	G_1_ (%)	S (%)	G_2_/M (%)	*n*
0	70.4 ± 2.2	15.5 ± 0.6	13.6 ± 2.4	7
0.1	68.8 ± 3.8	17.4 ± 2.1	14.2 ± 2.8	4
1	70.4 ± 3.6	15.9 ± 1.4	14.0 ± 2.6	4
10	69.6 ± 2.1	15.5 ± 1.2	15.2 ± 1.1	5
100	69.9 ± 2.9	15.4 ± 1.8	15.0 ± 1.3	5

### 2.6. Effects of MYZ on ROS

**Figure 7:**
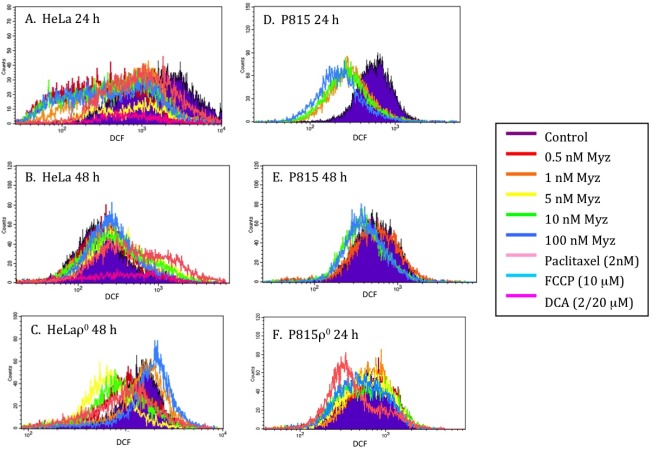
Effects of MYZ on reactive oxygen species (ROS) as determined by dichlorofluorescein (DCF) fluorescence. Representative histograms of fluorescence intensity of DCF, a marker of ROS production, are presented for HeLa (**A**, **B**) and P815 (**D**, **E**) cells 24 h and 48 h after addition of MYZ. Paclitaxel, a microtubule-stabilizing agent, was used as a negative control and dichloroacetate (DCA) as a positive control. DCF in HeLaρ^0^ at 48 h (**C**) and P815ρ^0^ at 24 h (**F**) are also presented. The geometric means of the fluorescence are presented in Table S1 of the Supplementary Data.

DCF (2,7-dichlorofluorescein) [17] was used to determine whether MYZ affected the production of reactive oxygen species (ROS) (Figure 7). After 24 h treatment with MYZ, decreased ROS levels were seen in both HeLa and P815 cells (Figure 7A,D) compared with untreated control cells, but by 48 h, the DCF response had returned to control levels (Figure 7B,E). With HeLaρ^0^ at 48 h (Figure 7C) and P815ρ^0^ at 24 h (Figure 7F), some lesser shifts in DCF fluorescence were also observed. Dichloroacetate (DCA) was chosen as a positive control for increasing ROS in the cells; however, it proved to be too toxic to the cells at the two concentrations tested (20 μM and 2 μM).

### 2.7. MYZ Activity Range in Different Cell Lines and Dependence on Mitochondrial Function

The mode of action of MYZ remained elusive until recently when Morgan *et al.* reported it to be a MET complex I inhibitor as well as an HIF-1 inhibitor [5,6]. These authors reported a large range in sensitivity of different cell lines to growth inhibition by MYZ, with the T47D breast cancer cell line being the most sensitive and another breast cancer cell line MDA-MB-231 being the least sensitive. McLaughlin [11] has also reported highly selective activity of acetogenins, potent complex I inhibitors, in different cell lines. When screened against the NCI 60 cell line panel, MYZ showed potent activity against the small and non-small lung cancer cell lines DMS114 (13 nM IC_50_) and NCI-H23 (250 nM IC_50_); however, there was a broad range of activity in other cell lines from 0.1–100 μM [4]. In HCT-116 colon cancer cells, MYZ had an IC_50_ of 3.8 nM [18]. In our study, MYZ also showed a broad range of activity in different cancer cell lines. In another NCI study of MET complex 1 inhibitors [10], leukemic cell lines such as K562 were particularly sensitive, yet in our study, although splenic T cells were very sensitive, the leukemic cell lines HL-60 and Jurkat showed a high level of resistance to MYZ.

The nanomolar response in sensitive cells suggests a direct link between NADH flux and MET, a principal site of NADH oxidation in respiring cells. To investigate this link, a panel of ρ^0^ cells was used. These cells lack mitochondrial DNA, and therefore have defective electron transport function at complexes I, III, and IV as well as having a non-functional cytochrome oxidase (complex V, [15]). Relative to MYZ-sensitive parental cells (0.36–14 nM IC_50_ range), ρ^0^ cells for these same cell lines had low sensitivity to MYZ in the MTT assay with IC_50_ values in the range of 26–78 μM. This amounts to a 2500–217,000-fold difference in MTT sensitivity between parental and ρ^0^ cells. With HL-60 cells, on the other hand, the IC_50_ values were similar between the parental (12 μM) and HL-60ρ^0^ (26 μM) cells. Thus, having functional MET made little difference to the effect of MYZ in this cell line. It was therefore concluded that MET was not necessary for the micromolar MYZ sensitivity seen in those cells. 

In a preliminary test with rotenone (Figure S3 of Supplementary Data), MYZ-insensitive HL-60 cells gave IC_50_ values of 0.41 μM for rotenone in wild type cells and 0.39 μM in HL-60ρ^0^ cells. Thus, the independence of mitochondrial function was similar for both complex I inhibitor drugs. It is possible that resistant cell lines that normally use oxidative phosphorylation to generate ATP, such as HL-60, are able to shift their metabolism to aerobic glycolysis in the presence of a complex I inhibitor like MYZ or rotenone. A shift to aerobic glycolysis, commonly associated with cancer cells, is referred to as the Warburg effect. In a study by Xu *et al. *[19], a mitochondrial-deficient clone of HL-60 cells (HL-60/C6F) showed enhanced inhibition of cellular ATP levels in the presence of the glycolytic inhibitor 3-bromopyruvate at concentrations of 50–100 μM, indicating that when mitochondrial function is impaired, the cells can alter their metabolism toward aerobic glycolysis. At a higher concentration of 300 μM 3-bromopyruvate, however, both HL-60 and HL-60/C6F cells were completely depleted of ATP and underwent apoptosis. 

Our findings with mitochondrial DNA-free cells are novel and suggest that the nanomolar effects of MYZ require MET function, but that MET function is not sufficient for these effects, since the insensitive cell lines were unaffected at nanomolar concentrations of MYZ, yet have normal mitochondrial function. Thus, some other factor or factors not present in insensitive cells appears to be required for the nanomolar effects of MYZ on MET. The nature of these MYZ-priming events and the extent to which they are involved with other complex I inhibitors is at present unknown. 

### 2.8. MYZ Action on Metabolism, Cell Cycle, and Cell Viability

Although MTT dye reduction assays are often referred to as cell proliferation assays, the assay primarily measures cytosolic NAD(P)H oxidoreductases [20,21] and therefore is more correctly a measure of cellular metabolic activity. Using this assay, we showed that MYZ had no effect on MTT responses of sensitive cells until at least 12 h exposure, with an apparent threshold effect at about 0.1 nM MYZ in P815 cells. This contrasts with uncouplers such as FCCP that enhance MET and oxygen consumption within seconds. MET complex inhibitors, like rotenone and myxothiazol, azide and cyanide, also act within minutes to prevent NADH oxidation [22]. Hence, MYZ appears to be an atypical MET complex I inhibitor in its kinetics of action. The reasons for the delay in MYZ action could be due to a slow uptake into the cell, its likely membrane localization, or the need to generate an active metabolite before it can work on its target. In the study by Morgan *et al.* [6], using both intact and digitonin-permeabilized cells, MYZ, like rotenone, caused an immediate decrease in oxygen consumption by the cells. Just how this metabolic decrease from inhibition of complex I relates to actual cell growth inhibition, as measured in this study, is not known. In human CEM leukemic cells, McLaughlin [11] reported a 2 or 3 day lag period before significant depletion (up to 76% depletion) of ribonucleotide triphosphates occurred following addition of the acetogenin bullatacin.

No cell cycle block was observed in the sensitive HeLa cells after 24 or 48 h exposure, even at MYZ concentrations as high as 20 μM. This effect was not specific to HeLa cells since the non-sensitive HL-60 cells also failed to show the G_2_/M arrest seen with rotenone. These results are consistent with a generalized metabolic effect of MYZ in sensitive cells rather than a specific effect on different phases of the cell cycle. The results are opposite to that of the complex I inhibitor rotenone, which arrests cells in G_2_/M of the cell cycle [23,24]. 

In MYZ sensitive cells, the cells remained viable at concentrations of MYZ that extensively reduced metabolism. At higher micromolar concentrations, the MTT response correlated with loss of cell viability in both insensitive cells and in sensitive cells that have a biphasic response to the compound. This introduced the possibility that a second mode of action was involved at these higher concentrations in both types of cells. The second target may only be affected at high concentrations of MYZ because of a low sensitivity binding site, or alternatively, because MYZ is being metabolized to another bioactive compound that reaches its threshold concentration for the second target only at high concentrations. It seems unlikely that increases in ROS levels are involved in the cytotoxicity, since the DCF signal was decreased in HeLa and P815 cells after 24 h treatment with MYZ. It is surprising that MYZ reduced ROS levels in these two cell lines, given that complex I inhibitors like rotenone generally increase O_2_^.−^, the predominant ROS in cells [10]. 

### 2.9. Possible Mechanisms of MYZ Action

#### 2.9.1. Lack of Correlation to NQO1 Reductase

To understand the huge difference between IC_50_ values for MYZ between sensitive and insensitive cells, other differences between the cell lines were considered. For example, NAD(P)H:quinone oxidoreductase 1 (NQO1) levels vary substantially between different cell lines. NQO1 is an oxidoreductase that is involved in detoxifying quinones and also plays a role in the cellular response to oxidative stress [25,26]. There was no correlation, however in the different cell lines between NQO1 levels and sensitivity to MYZ. Thus, HL-60, an insensitive cell line, and P815 and B16, two sensitive cell lines, all have very low NQO1 levels; whereas, HeLa and MDCK, two other sensitive cell lines, have high levels of NQO1 ([27,28], and unpublished results). 

#### 2.9.2. Differences between MYZ and Rotenone

MYZ and rotenone are both MET complex I inhibitors, yet their activity profiles vary considerably. (i) There is a clear lack of G_2_/M arrest with MYZ, despite a reported G_2_/M arrest by rotenone [23,24]. The explanation for this is that rotenone, in addition to its complex 1 inhibitory effect, also binds tubulin and inhibits its polymerization. This secondary, direct effect on tubulin and subsequent mitotic block by rotenone has been well established [29]. The IC_50_ for rotenone in inhibiting proliferation in HeLa cells was 0.2 ± 0.1 μM, a concentration that also effectively depolymerized spindle microtubules and blocked mitosis [29]. (ii) ROS is decreased by MYZ compared with rotenone which increases ROS. (iii) in contrast to rotenone, MYZ has a long, 12 h lag period before metabolism is inhibited in the MTT assay. No such lag is seen in the inhibition by MYZ of oxygen consumption in non-permeabilized T47D cells [6]. MYZ is a small lipophilic molecule that should rapidly partition in membranes. Its movement to intracellular membrane systems, including mitochondrial membranes, may be slow compared with rotenone, resulting in delayed activation.

#### 2.9.3. Dual Target Mechanism and Future Directions

The presence of both sensitive and insensitive cell lines and biphasic effects in some sensitive cells can be explained by a two-target mechanism for MYZ. At low nanomolar concentrations, the drug or metabolite may interact with a metabolic target, either directly or indirectly affecting complex I, hence the loss of the sensitivity to MYZ in ρ^0^ cells. When higher concentrations are reached, a second target may be affected that causes cell death. Thus, the first target appears to be metabolic, since interaction at these concentrations does not kill the cells but has a cytostatic effect. This high sensitivity target is dependent on mitochondrial function since ρ^0^ cells are not susceptible to low nanomolar MYZ concentrations. Although these data support complex I being the primary target in these cells, they do not explain the insensitivity of some cells with intact mitochondria and functional MET, such as HL-60, LN18, and Jurkat [30]. It is possible that these MYZ-insensitive cells are better able to alter their metabolism to aerobic glycolysis to overcome the action of a MET complex I inhibitor. The second, low sensitivity, micromolar target is not affected by loss of MET, as can be seen by similar MTT responses by HL-60 and HL-60ρ^0^ cells to MYZ (and to rotenone, Figure S3 of Supplementary Data). Inhibition via this second target has cytotoxic effects on cells (Figure 4), indicating a distinct non-mitochondrial mechanism of action. 

## 3. Experimental Section

### 3.1. MYZ Extraction and Purification

Methanolic extracts of the marine sponge *Cacospongia mycofijiensis*, collected from ‘Eua, Tonga were fractionated to generate MYZ in greater than 95% purity. All spectroscopic data, including ^1^H and ^13^C NMR (Figure S1 of Supplementary Data), optical rotation and high-resolution mass spectrometry were in accordance with previously reported accounts of MYZ [1,3]. The NMR spectra (CDCl_3_, 600 MHz) were obtained using a Varian DirectDrive spectrometer equipped with a triple resonance HCN cryogenic probe. High-resolution positive-ion mass spectra were recorded on a Waters Q-TOF Premier™ Tandem Mass spectrometer. Optical rotations were performed using a Rudolph Autopol II polarimeter. Normal-phase column chromatography was carried out with silica gel. Reversed-phase column chromatography was achieved using Supelco Diaion HP20 poly(styrene-divinylbenzene) (PSDVB) chromatographic resin. HPLC was performed using a Rainin Dynamax SD-200 solvent delivery system, with UV detection using a Varian ProStar 335 photodiode array detector. Solvents used for flash normal- and reversed-phase column chromatography were of HPLC or analytical grade quality. All other solvents were purified by glass distillation. Solvent mixtures are reported as % (v/v) unless otherwise stated. *Cacospongia mycofijiensis* sponge specimens were stored at −20 °C until required for extraction. Frozen sponge (21.0 g) was extracted in MeOH (2 × 100 mL) and loaded onto PSDVB. The loaded extracts were eluted with: (i) 30% Me_2_CO/H_2_O, (ii) 75% Me_2_CO/H_2_O and (iii) Me_2_CO. A portion of the 75% Me_2_CO/H_2_O fraction was fractionated further on PSDVB (30–100% Me_2_CO/H_2_O). The 60–70% Me_2_CO/H_2_O fractions were further purified using silica gel (CH_2_Cl_2_–EtOAc). The 10–20% EtOAc/CH_2_Cl_2_ fractions were finally purified using HPLC (DIOL, 5 μm, 4.6 × 250 mm, 10% IPA/*n*-hexane) to yield mycothiazole (5.7 mg, *t_R_* = 5.5 min) as a colorless oil with the following properties: [α]_D_^20^ −19.3° (*c* 0.93, CHCl_3_); HRESIMS [M + H]^+^, observed *m*/*z* 405.2215, calculated *m*/*z* 405.2212 for C_22_H_33_N_2_O_3_S, Δ = +0.7 ppm.

### 3.2. Cell Culture

Cell lines used in this study included murine splenic T-cells, adherent cell lines of human (HeLa cervical carcinoma, HeLa S3 semi-adherent clone, 143B osteosarcoma, LN18 glioblastoma), mouse (4T1 breast carcinoma, B16 melanoma), and dog origin (MDCK kidney epithelial), and non-adherent cell lines of human (HL-60 promyelocytic leukemia, Jurkat T-cell lymphoma) and mouse origin (P815 mastocytoma, RAW264.7 monocyte/macrophage). Cell lines were obtained from the following sources: HeLa (Professor Anthony Braithwaite, University of Otago), HeLa S3 (Dr. Alfons Lawen, University of Melbourne), 143B and 143Bρ^0^ (Dr. Mike Murphy, University of Otago), P815 (Dr. John Marbrook, University of Auckland), HL-60 (Dr. Graeme Findlay, University of Auckland), Jurkat (Dr. Thomas Bäckström, Malaghan Institute, Wellington), LN18, 4T1, B16, MDCK, and RAW264.7 (ATCC, Manassas, VA). The parent lines are all phenotypically stable and exhibit properties characteristic of their cell or tissue of origin. HL-60 is regularly tested in our laboratory for respiratory burst activity and differentiation into neutrophils (retinoic acid) or monocytes (PMA or vitamin D_3_). Four of the five ρ^0^ cell lines lacking mitochondrial DNA (HL-60ρ^0^, HeLaρ^0^, P815ρ^0^, and B16ρ^0^) used in this study, with the exception of 143Bρ^0^, were generated in our laboratory by prolonged culture of the parental cell lines in the presence of ethidium bromide, followed by culture in its absence [14]. These phenotypically stable cell lines were auxotrophic for uridine and pyruvate. All ρ^0^ cells were tested at regular intervals for loss of MET function by FCCP resistance of WST-1 reduction, and by PCR for absence of mitochondrially-encoded cytochrome b. 

The standard culture medium consisted of RPMI-1640 medium supplemented with 5–10% fetal calf serum, 2 mM glutamate, 25 μg/mL penicillin, 25 μg/mL streptomycin, 50 μg/mL uridine, and 1 mM pyruvate. Murine B16 cells, however, were cultured in complete medium (cIMDM) containing Iscove’s modified Dulbecco’s medium (IMDM), 2 mM Glutamax^®^, 10% fetal calf serum, 100 U/mL penicillin, 100 µg/mL streptomycin with 50 μg/mL uridine and 1 mM pyruvate. RAW264.7 cells and CD4/CD8 T cells isolated from the mouse spleen were cultured in a DMEM-based complete T cell medium containing 10% FCS and other supplements as previously described [31]. All cells were grown at 37 °C in an atmosphere of 5% CO_2_ in air and high humidity. Adherent cell lines were passaged using TrypLE™ Express (Invitrogen) to detach the cells, washed with medium, and then passaged into fresh medium.

### 3.3. Cell Metabolism Assay

An MTT cell proliferation assay [20,21] was used to measure cell metabolism. Cells reduce the yellow 3-(4,5-dimethylthiazol-2-yl)-2,5-diphenoyltetrazolium bromide (MTT) dye to blue formazan crystals in a manner dependent on intracellular reduced pyridine nucleotides, and therefore the assay is a measure of cell metabolism. Cells were seeded at a density of 1 × 10^4^ cells/well in a 96-well plate and treated with varying concentrations of MYZ for different lengths of time. MTT solution (20 μL of 5 mg/mL in PBS) was added to each well, and after 2 h incubation, 100 μL of solubilizer (10% SDS, 45% *N*,*N*-dimethylformamide) was added to lyse the cells and dissolve the blue crystals. The absorbance was measured at 570 nm using a multi-well plate reader. Absorbances were then used to generate concentration-response curves and to calculate IC_50_ values (the concentration at which MTT reduction is 50% of a non-treated control) using SigmaPlot software.

### 3.4. ^3^H-Thymidine Uptake

Cells were seeded in 96-well plates at a density of 1 × 10^5^ cells/mL and treated with MYZ. For the last 8 h of culture, 1 μCi ^3^H-thymidine was added to each well, and the plates were frozen to stop cell proliferation. After thawing, the cells in each well were transferred to filters using a cell harvester (Tomtec, Hamden, CT) and the radioactivity determined in a liquid scintillation counter (Wallac MicroBeta TriLux). 

### 3.5. CFSE Cell Proliferation Assay

In primary cultures of splenic T cells, a carboxyfluorescein succinimidyl ester (CFSE) proliferation assay was used to determine the IC_50_ for growth inhibition, as previously described [31]. Briefly, splenocytes were isolated from female C57BL/6 mice and stained with 1 µM CFSE. Cells were then treated with different concentrations of MYZ in the presence or absence of Con A (3 µg/mL; Sigma Chemical Co.) for 48 h. The cells were then harvested and stained with fluorescently-labelled antibodies (anti-CD4-cychrome c and anti-CD8-PE; BD Biosciences) or isotype controls, according to the manufacturer’s recommendations, and analyzed by flow cytometry using a FACScan (Becton Dickinson). A total of 10,000 events were collected for each assay, and the data were analyzed by CellQuest Pro software (Becton Dickinson).

### 3.6. Trypan Blue Assay for Cell Viability

A trypan blue dye exclusion assay was used to determine cell viability. Cells (1 × 10^5^/mL) were cultured with varying concentrations of MYZ and then stained with trypan blue and counted in a hemocytometer.

### 3.7. Cell Cycle Analysis

Flow cytometric analysis of cell DNA content following staining with propidium iodide (PI) was used to assess progression through the cell cycle. Cells were seeded at 1 × 10^5^ cells/well in 24-well plates and incubated with MYZ for various times. The cells were detached with 50 μL TrypLE^TM^ Express, washed in PBS, and then incubated with 250 μL of PI solution (0.05 mg/mL PI, 0.1% sodium citrate, 0.1% Triton X-100 in PBS). Samples were scanned and analyzed on a FACScan flow cytometer with BD Cell Quest Pro software.

### 3.8. Measurement of Cellular ROS

Mitochondrial ROS was measured by flow cytometry using changes in fluorescence of dichlorofluorescein (DCF). Briefly, cells were prepared as for cell cycle analysis, then 230 μL of 10 μM DCF in PBS was added and left for 30 min. PI (20 μL of 100 μg/mL) was also added to allow gating on only live, PI-negative cells. The samples were then scanned on a FACScan flow cytometer with BD Cell Quest Pro software.

## 4. Conclusions

The main finding of this study was that MYZ requires mitochondrial function to exert its nanomolar effects, providing support for its action as a MET complex I inhibitor, but that some cell lines display a biphase response or are resistant to the action of the compound. Studies on isolated mitochondria and individual MET complex activities may better define the cellular mechanism of action of MYZ. It would be interesting to compare the effect of MYZ on mitochondrial oxygen consumption in sensitive and insensitive cell lines. This would clarify whether differences in sensitivity to the drug are due to drug uptake/efflux mechanisms or different targets. It would also be interesting to explore the secondary cytotoxic mechanism of action of MYZ that occurs in cells at high, micromolar concentrations.

## Supplementary Files

Supplementary File 1: PDF-Document (PDF, 322 KB)
