# Supplementary material for: Mitochondrial Genome-Knockout Cells Demonstrate a Dual Mechanism of Action for the Electron Transport Complex I Inhibitor Mycothiazole
Source: Mar Drugs. 2012 Apr 16;10(4):900–17. doi: 10.3390/md10040900 (PMC3366682; doi:10.3390/md10040900)

Supplementary Data

Mitochondrial Genome-Knockout Cells Demonstrate a Dual Mechanism of Action for the Electron Transport Complex I Inhibitor Mycothiazole

Kirsten J. Meyer, A. Jonathan Singh, An S. Tan, Alanna Cameron, Dora C. Leahy, David O'Sullivan, Praneta Joshi, Anne C. La Flamme, Peter T. Northcote, Michael V. Berridge and John H. Miller

Figure S1. Mycothiazole ^{1}H (A) and ^{13}C (B) NMR spectra. NMR spectra (CDCl3, 600 MHz) were obtained using a Varian DirectDrive spectrometer equipped with a triple resonance HCN cryogenic probe. Peaks at dH 5.30 and dC 53.8 are residual dichloromethane. Peaks at dH 1.25 and dC 29.8 are fatty acid residue in the preparation.

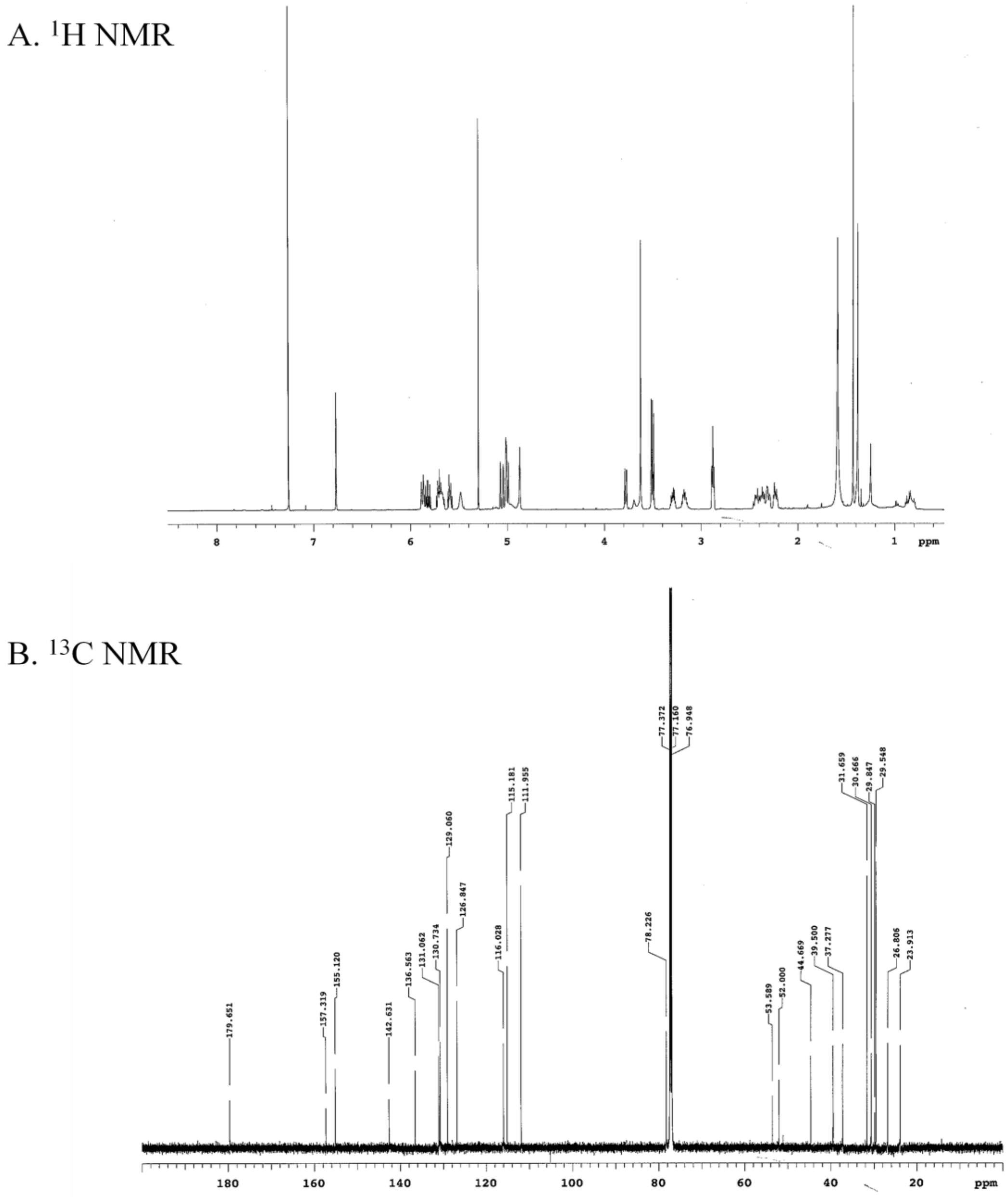

Figure S2. Percentage of HL-60 cells in different phases of the cell cycle after treatment for 16 h with MYZ at concentrations ranging from 0.1 to 50 mM (n = 3 preparations; values are mean ± SEM).

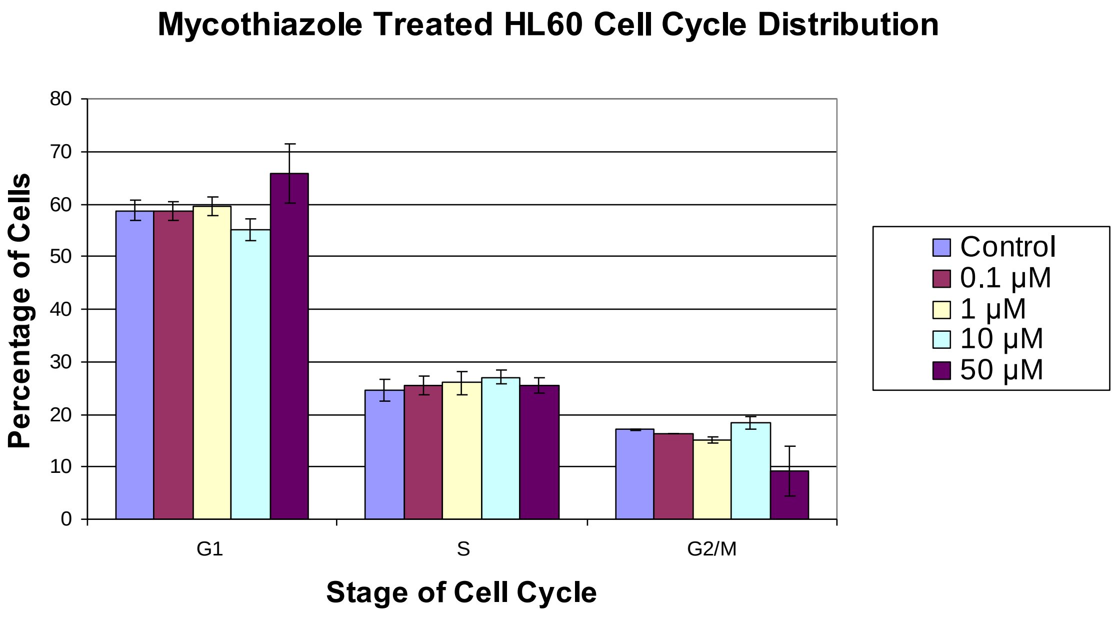

Table S1. Geometric means of the DCF fluorescence values for data of Figure 7.

Cell Line	Time (h)	0 nM MYZ	0.5 nM MYZ	1 nM MYZ	5 nM MYZ	10 nM MYZ	100 nM MYZ	2 nM Paclitaxel
HeLa	24	1405	290	548	633	372	379	904
HeLa	48	203	224	252	343	293	262	469
P815	24	381		282		176	237	
P815	48	508		598		516	491	
HeLaρ^0	48	1279	973	1393	646	849	1702	933
P815ρ^0	24	553	614	630	583	552	517	407

Figure S3. Effect of rotenone and MYZ on proliferation of HL-60 and HL-60r^{0} cells. Representative graphs of 48 h MTT assays for rotenone and MYZ are presented (n = 2 independent experiments). The average IC_{50} values from the two experiments are presented on each graph.

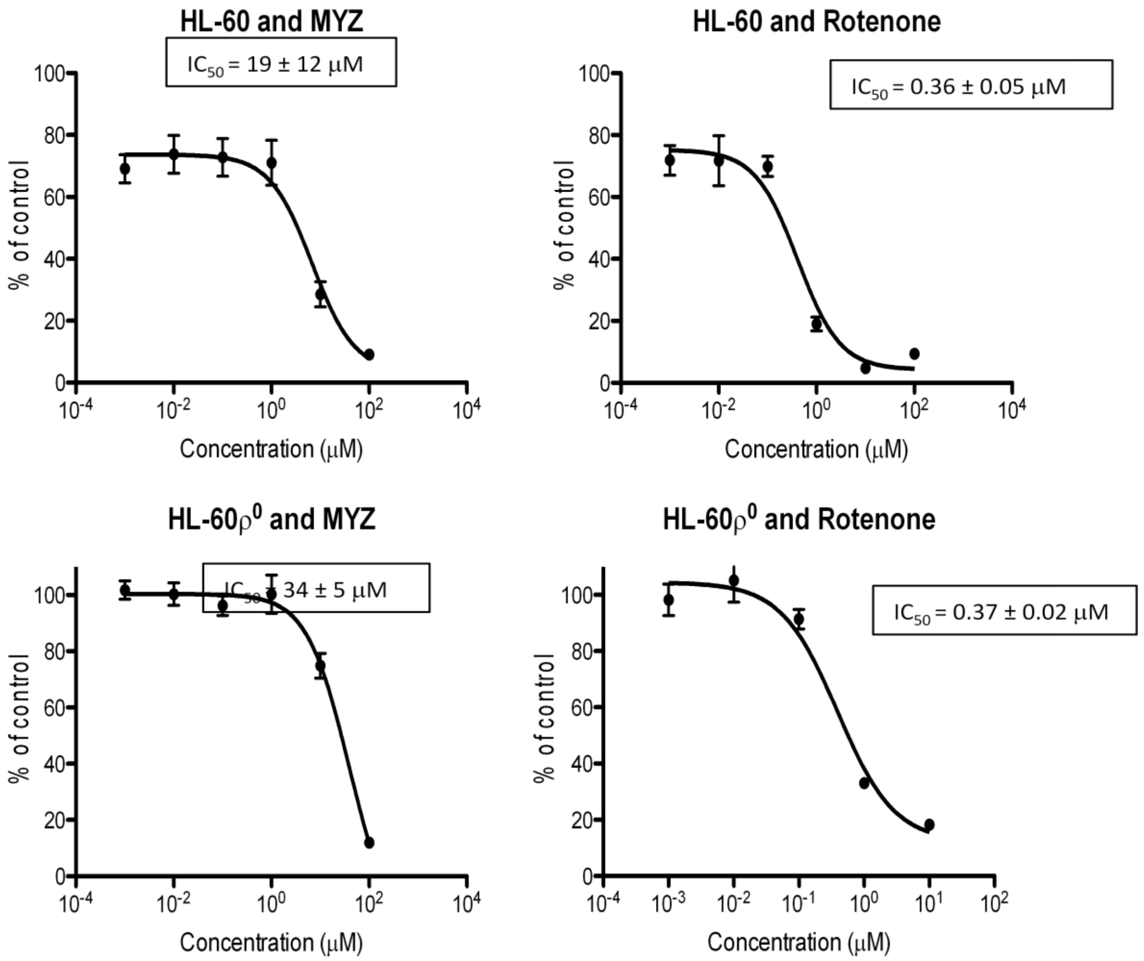

Supplement: Supplementary File 1: — PDF-Document (PDF, 322 KB) [file marinedrugs-10-00900-s001.pdf]
